# 铁缺乏症患者缺铁程度对口服铁剂吸收的影响

**DOI:** 10.3760/cma.j.issn.0253-2727.2021.05.009

**Published:** 2021-05

**Authors:** 靖 胡, 向荣 胡, 小霞 李, 旭 刘, 夏婉 杨, 东蕊 关, 京倩 刘, 凤奎 张

**Affiliations:** 中国医学科学院、北京协和医学院血液病医院（中国医学科学院血液学研究所），实验血液学国家重点实验室，国家血液系统疾病临床医学研究中心，天津 300020 State Key Laboratory of Experimental Hematology, National Clinical Research Center for Blood Diseases, Institute of Hematology & Blood Diseases Hospital, Chinese Academy of Medical Sciences & Peking Union Medical College, Tianjin 300020, China

**Keywords:** 缺铁性贫血, 口服铁剂吸收试验, 铁吸收, 铁调素

## Abstract

**目的:**

研究铁缺乏症患者缺铁程度对口服铁剂吸收的影响。

**方法:**

纳入2018年7月至2020年6月于中国医学科学院血液病医院贫血中心门诊就诊并确诊铁缺乏症的非妊娠期女性患者37例，以13例健康查体女性为正常对照，分析缺铁性贫血（IDA）、缺铁性红细胞生成/储存铁不足（IDE/ID）患者及正常对照者Hepcidin水平并进行口服铁剂吸收试验（OIAT），比较服铁后2 h血清铁（C2）与基线血清铁（C0）差值。

**结果:**

IDA、ID/IDE、正常对照组Hepcidin中位数分别为4.9（2.17～32.86）、26.98（11.02～49.71）、69.89（42.23～138.96）µg/L（*P*<0.001），IDA组Hepcidin低于ID/IDE组（校正后*P*＝0.005）和正常对照组（校正后*P*<0.001），但ID/IDE组与正常对照组差异无统计学意义（校正后*P*＝0.220）；OIAT IDA、ID/IDE及正常对照组C2–C0平均值分别为（35.30±21.68）、（37.90±14.06）、（23.57±10.14）µmol/L，差异无统计学意义（*P*＝0.130）。多重线性回归分析显示C0、血清铁蛋白（SF）、可溶性转铁蛋白受体（sTFR）和HGB是铁缺乏症患者Hepcidin的独立影响因素，Hepcidin＝−31.842−0.642*C0+2.239*SF+1.778*sTFR+0.365*HGB−0.274*网织红细胞血红蛋白含量（RET-HB）。未发现C2–C0的独立影响因素。

**结论:**

铁缺乏程度影响口服铁剂吸收，胃肠功能正常者铁缺乏越重，口服铁剂吸收越多，ID/IDE较IDA铁吸收减缓。Hepcidin较OIAT更能区分不同铁缺乏程度的口服铁吸收。

## Abstract (en)

**Objective:**

To study the effect of iron deficiency level for oral iron absorption in iron deficient patients.

**Methods:**

37 non-pregnant female patients who were diagnosed with iron deficiency and 13 healthy females who completed their physical examination at the outpatient department of the Anemia Center of the Institute of Hematology & Blood Diseases Hospital from July 2018 to June 2020 were included. Hepcidin and C2–C0 of oral iron absorption test were analyzed in different iron deficiency and serum ferritin level.

**Results:**

The median of Hepcidin in IDA, ID/IDE and healthy control group were 4.9（2.17–32.86）, 26.98（11.02–49.71）and 69.89（42.23–138.96）µg/L（*P*<0.001）, respectively. Hepcidin level of IDA group was lower than that of ID/IDE group （adjusted *P*＝0.005） and healthy control （adjusted *P*<0.001）. Hepcidin level of ID/IDE group had no significant difference compared with healthy control（adjusted *P*＝0.22）. The mean of C2–C0 in IDA, ID/IDE and healthy control group were（35.30±21.68）, （37.90±14.06） and （23.57±10.14） µmol/L（*P*＝0.130）, respectively. Multilinear regression analysis showed C0, SF, sTFR and HGB were independent factors for Hepcidin in iron deficient patients, with an equation of Hepcidin＝−31.842−0.642*C0+2.239*SF+1.778*sTFR+0.365*HGB−0.274*RET-HB. We didn't find independent factor of C2–C0.

**Conclusion:**

The degree of iron deficiency had an effect on oral iron absorption. Patients of ID/IDE group absorbed iron more slowly than patients of IDA group. Iron deficient patients with normal gastrointestinal function absorbed more iron by oral administration when they were in a more serious iron deficient stage. Hepcidin was a better parameter to distinguish iron absorption level among different iron deficient patients than C2–C0 of oral iron absorption test.

铁缺乏症由摄入不足、吸收不良和慢性失血等引起[1]。根据机体铁缺乏程度的不同，铁缺乏症可分为储存铁不足（ID）、缺铁性红细胞生成（IDE）和缺铁性贫血（IDA）三个阶段。治疗IDA最常用的方法为口服补铁，为提高铁剂吸收效率与生物利用度，近年来倾向于研发新型口服铁剂，改善胃肠道铁吸收状态和改变给药剂量、方式[2]–[3]，而对缺铁状态本身对铁吸收的影响少有研究。口服铁剂吸收试验（OIAT）常用于评估胃肠道铁吸收以预测口服补铁疗效，口服铁剂2 h血清铁浓度（C2）较空腹基线值（C0）上升小于17.857 µmol/L（1000 µg/L）提示口服铁吸收不良[4]–[5]。铁调素（Hepcidin）与铁通道蛋白（FPN）结合使后者内化降解，减少胃肠道铁吸收，也用于预测口服铁吸收不良。本研究中，我们纳入无胃肠疾患仅因摄入不足或失血过多引起的单纯铁缺乏患者，对不同程度铁缺乏患者胃肠道铁吸收进行了研究。

## 病例与方法

一、病例资料

纳入2018年7月至2020年6月于中国医学科学院贫血诊疗中心门诊就诊并确诊的非妊娠期女性单纯铁缺乏症患者37例，其中IDA 28例，IDE 2例，ID 7例（3例为IDA治疗后恢复至ID）。以同期13例健康查体女性为正常对照。所有患者均仔细询问病史，进行血常规、血清铁蛋白（SF）、血清铁、转铁蛋白饱和度（TSAT）、可溶性转铁蛋白受体（sTfR）和Hepcidin检测。16例铁缺乏症患者［IDA 9例，IDE 1例，ID 6例（1例为IDA治疗后恢复至ID）］及11例正常健康女性进行了OIAT。本研究方案获我院伦理委员会批准（批件号：IIT2019003-EC-1），所有研究对象均签署知情同意书。

二、诊断标准[6]

1. IDA：符合IDA诊断标准的初诊患者：①小细胞低色素性贫血：HGB<110 g/L，平均红细胞体积（MCV）<80 fl，平均血红蛋白浓度（MCHC）<320 ng/L，红细胞形态可有明显低色素表现。②空腹血清铁<10.7 µmol/L（60 µg/L），TSAT<0.15。③SF<20 µg/L。

2. IDE：①新诊断的IDE患者或原IDA治疗后进入IDE期的患者；②HGB≥110 g/L；③TSAT<0.15或MCV<80 fl、MCHC<320 ng/L，sTfR>2.25 mg/L，SF<20 µg/L。

3. ID：①HGB≥110 g/L，MCV、MCHC正常；②TSAT≥0.15，sTfR≤2.25 mg/L，SF<20 µg/L。

三、排除标准

凡符合以下任何一项标准的病例不纳入研究：①患有AA、阵发性睡眠性血红蛋白尿症、骨髓增生异常综合征等其他血液病或其他肿瘤；②疑诊血红蛋白病、地中海贫血，或有相关疾病家族史；③患有明确胃肠道疾病或有胃肠道手术史，或因反酸、上腹痛等不适近期口服奥美拉唑、雷贝拉唑等抑酸药；④半年内使用过静脉铁剂，2周内口服过任意种类的铁剂[7]；⑤1个月内有感染或输血史；⑥患有慢性肝炎、肾功能不全。

四、Hepcidin检测

使用武汉华美生物工程有限公司人Hepcidin ELISA Kit（CSB-E13062h）试剂盒检测血浆Hepcidin，根据操作说明测定结果，为保证检测结果准确性标准品及所有样品均设复孔。

五、铁代谢相关指标及OIAT检测

铁代谢相关指标检测由本院检验科生化室按照标准操作规程完成，血清铁采用美国Beckman Coulter公司AU480全自动生化分析系统检测，SF采用化学发光法检测，sTfR采用免疫比浊法检测。2例正常对照分别于空腹，服用硫酸亚铁0.3 g（含铁60 mg）后1.5、2、3、4 h检测血清铁浓度，C2较C0均升高明显（表1），与文献报道时间点相符[4]–[5]，故采用C2作为参比浓度。比较不同程度铁缺乏患者及正常对照的C2及C2–C0。

**表1:** 2例正常对照空腹至服铁后4 h血清铁（µmol/L）

例号	0 h	1.5 h	2 h	3 h	4 h
正常对照1	11.84	28.60	32.32	31.42	29.74
正常对照2	19.78	41.57	48.05	55.40	57.85

六、统计学处理

采用SPSS 26.0统计软件进行数据分析，正态分布资料以均数±标准差描述，两组间比较采用独立样本*t*检验，多组间比较采用单因素方差分析，两两比较采用LSD法进行事后检验；非正态分布资料以中位数（最小值～最大值）描述，两组间比较采用独立样本Mann-Whitney *U*检验，多组间比较采用Kruskal-Wallis *H*检验，两两比较使用Bonferroni法校正。连续变量进行Spearman相关分析，有统计学意义的相关因素纳入多重线性回归进行分析。*P*<0.05为差异有统计学意义。

## 结果

1. 基线及铁代谢参数特征：正常对照组平均年龄32.31±7.25岁，铁缺乏症组平均年龄38.32±9.33岁，两组年龄、身高、体重、BMI差异均无统计学意义。铁缺乏症组中位HGB为87（56～143）g/L，中位WBC为4.84（3.24～7.94）×10^9^/L、中位网织红细胞血红蛋白含量（RET-HB）为19.5（13.4～33.8）pg、中位MCV 71.4（60.4～98.9）fl、中位平均血红蛋白含量（MCH）为20.9（15.7～32.4）pg、中位MCHC为288（258～341）ng/L，均明显低于正常对照（*P*值均<0.05），PLT、网织红细胞比例、网织红细胞绝对计数两组差异无统计学意义。铁缺乏症组中位SF为3.0（1.1～18.1）µg/L，显著低于正常对照组的35.1（21.3～63.6）µg/L（*P*<0.001），铁缺乏症组中位sTfR为3.87（1.16～10.20）mg/L，显著高于正常对照组的1.09（0.81～1.50）mg/L（*P*<0.001）。IDA组转铁蛋白为（3.37±0.57）g/L，高于ID/IDE组［（2.67±0.38）g/L，*P*＝0.003］和正常对照组［（2.44±0.43）g/L，*P*<0.001］（表2）。

2. Hepcidin水平比较：Hepcidin在IDA、ID/IDE、正常对照组中位数分别为4.9（2.17～32.86）、26.98（11.02～49.71）、69.89（42.23～138.96）µg/L（*P*<0.001）。IDA组Hepcidin低于ID/IDE组（校正后*P*＝0.005）和正常对照组（校正后*P*<0.001）；ID/IDE组与正常对照组血浆Hepcidin水平差异无统计学意义（校正后*P*＝0.220）。将IDA组28例患者以SF中位数（2.5 µg/L）分为二组，SF≤2.5 µg/L组Hepcidin低于SF>2.5 µg/L组［4.07（2.17～8.10）µg/L对8.08（4.22～32.86）µg/L，*P*＝0.001］。

3. 铁缺乏症患者OIAT结果：将表3。16例铁缺乏症患者（9例IDA、7例ID/IDE）和11例正常对照进行了OIAT检测。C2较C0明显升高，IDA、ID/IDE、正常对照C2分别为38.18±21.93、55.1±16.18和42±12.49 µmol/L（*P*＝0.147），分别为C0的11.9、3.32和2.45倍（*P*<0.001）。IDA、ID/IDE及正常对照组2 h TSAT（TSAT2）中位数分别为0.39（0.17～0.96）、0.93（0.48～0.96）和0.67（0.42～0.95）（*P*＝0.043），IDA组TSAT2低于ID/IDE组（校正后*P*＝0.038），其余各组间差异无统计学意义。

铁缺乏症组C2–C0明显较正常对照组更高（*P*＝0.044），IDA、ID/IDE和正常对照组C2–C0间差异无统计学意义（*P*＝0.130）。IDA及ID/IDE患者C2–C0平均值分别为（35.30±21.68）µmol/L和（37.90±14.06）µmol/L（*P*＝0.747），高于正常对照组C2–C0平均值（23.57±10.14）µmol/L（*P*值分别为0.111、0.073），差异均无统计学意义。

IDA、ID/IDE、正常对照组（C2–C0）/C0中位数分别为10.90（5.37～37.14）、2.32（0.78～3.90）、1.45（0.53～2.66）（*P*<0.001），ID/IDE组（C2–C0）/C0低于IDA组（校正后*P*＝0.037），但与正常对照组比较差异无统计学意义（校正后*P*＝0.407）。

4. 影响铁缺乏患者胃肠铁吸收的相关因素：铁缺乏症组患者Hepcidin与C0（*r*＝0.738，*P*<0.001）、SF（*r*＝0.880，*P*<0.001）、sTfR（*r*＝0.584，*P*<0.001）、HGB（*r*＝0.639，*P*<0.001）、RET-HB（*r*＝0.633，*P*<0.001）呈正相关，与C2（*r*＝0.147，*P*＝0.587）、C2–C0（*r*＝-0.097，*P*＝0.721）无明显线性相关性。进一步纳入多重线性回归分析，结果显示C0（*P*＝0.021）、SF（*P*<0.001）、sTFR（*P*＝0.035）和HGB（*P*＝0.001）为Hepcidin的独立影响因素（*R*^2^＝0.878，*P*<0.001），Hepcidin＝−31.842−0.642*C0+2.239*SF+1.778*sTFR+0.365*HGB−0.274*RET-HB。OIAT C2–C0与HGB、RET-HB、SF、sTfR相关性无统计学意义。

**表2:** 铁缺乏症患者及正常对照基线资料比较

指标	正常对照组（13例）	铁缺乏症组（37例）	*P*值
年龄（岁，*x*±*s*）	32.31±7.25	38.32±9.33	0.400
身高（m，*x*±*s*）	1.61±0.05	1.62±0.05	0.475
体重（kg，*x*±*s*）	59.35±8.08	60.03±7.78	0.790
BMI［kg/m^2^，*M*（范围）］	22.41（18.73～27.73）	22.66（17.22～72.50）	0.972
HGB［g/L，*M*（范围）］	138（124～147）	87（56～143）	<0.001
MCV［fl，*M*（范围）］	89.7（84.8～93.3）	71.4（60.4～98.9）	<0.001
MCH［pg，*M*（范围）］	30.4（27.6～31.4）	20.9（15.7～32.4）	<0.001
MCHC［ng/L，*M*（范围）］	336（318～345）	288（258～341）	<0.001
WBC［×10^9^/L，*M*（范围）］	6.31（4.16～10.96）	4.84（3.24～7.94）	0.002
PLT［×10^9^/L，*M*（范围）］	238（110～336）	267（118～534）	0.215
RET［％，*M*（范围）］	1.53（0.99～3.02）	1.33（0.63～3.43）	0.928
RET计数［×10^9^/L，*M*（范围）］	67.2（44.2～142.8）	56.9（28.4～143.0）	0.216
RET-HB［pg，*M*（范围）］	31.6（29.9～33.1）	19.5（13.4～33.8）	<0.001
C0［µmol/L，*M*（范围）］	19.59（11.33～24.67）	3.08（1.55～28.32）	<0.001
TSAT［*M*（范围）］	0.30（0.19～0.38）	0.04（0.02～0.39）	<0.001
Hepcidin［µg/L，*M*（范围）］	69.89（42.23～138.96）	6.93（2.17～49.71）	<0.001
SF［µg/L，*M*（范围）］	35.1（21.3～63.6）	3.0（1.1～18.1）	<0.001
Tf（g/L，*x*±*s*）	2.44±0.43	3.21±0.61	<0.001
sTfR［mg/L，*M*（范围）］	1.09（0.81～1.50）	3.87（1.16～10.20）	<0.001
sTfR/lgSF［*M*（范围）］	0.71（0.51～1.04）	8.34（0.97～117.65）	<0.001

注：BMI：身体质量指数；MCV：平均红细胞体积；MCH：平均血红蛋白含量；MCHC：平均血红蛋白浓度；RET％：网织红细胞比值；RET计数：网织红细胞绝对计数；RET-HB：网织红血红蛋白含量；C0：空腹血清铁浓度；TSAT：空腹转铁蛋白饱和度；Hepcidin：铁调素；SF：铁蛋白；Tf：转铁蛋白；sTfR：可溶性转铁蛋白受体

**表3:** 各铁缺乏症亚组及正常对照口服铁剂吸收结果比较

指标	正常对照（11例）	铁缺乏症（16例）	IDA（9例）	ID/IDE（7例）
C2（µmol/L，*x*±*s*）	42.00±12.49	45.59±20.89	38.18±21.93	55.10±16.18
C2–C0（µmol/L，*x*±*s*）	23.57±10.14	36.44±18.20	35.30±21.68	37.90±14.06
（C2–C0）/C0［*M*（范围）］	1.45（0.53～2.66）	5.79（0.78～37.14）	10.90（5.37～37.14）	2.32（0.78～3.90）
C2/C0［*M*（范围）］	2.45（1.53～3.66）	6.79（1.78～38.14）	11.90（6.37～38.14）	3.32（1.78～4.90）
C2/正常C0均值［*M*（范围）］	2.25（1.38～3.35）	2.12（0.59～4.31）	1.69（0.59～4.31）	3.33（1.60～3.93）
TSAT2［*M*（范围）］	0.67（0.42～0.95）	0.56（0.17～0.96）	0.39（0.17～0.96）	0.93（0.48～0.96）

注：IDA：缺铁性贫血；ID/IDE：储存铁不足/缺铁性红细胞生成；C2：口服0.3 g硫酸亚铁2 h后血清铁浓度；C0：空腹血清铁浓度；TSAT2：口服0.3 g硫酸亚铁2 h后转铁蛋白饱和度

## 讨论

Hepcidin-FPN轴在铁稳态调节，包括单核巨噬细胞铁循环再利用和胃肠铁吸收中起中心作用。主要由肝细胞分泌的短肽Hepcidin与目前已知唯一能输出铁的通道蛋白FPN互为配基，二者结合后FPN内化降解[8]。Hepcidin升高铁吸收下降，Hepcidin降低铁吸收升高[9]–[10]。

本研究结果表明在无胃肠道疾患和其他炎症性异常存在的单纯性铁缺乏患者，不同的缺铁状态Hepcidin降低程度也不同，提示其口服铁吸收的能力不同，与既往在不同程度缺铁孕妇中检测Hepcidin水平的结果一致[11]。IDA期血清铁、SF水平低下，与贫血引起的压力性红细胞生成所产生的ERFE共同抑制Hepcidin合成，故Hepcidin明显降低，口服铁剂能顺利通过FPN吸收[12]，铁吸收增加。IDE期贫血纠正，虽仍有缺铁性红细胞生成及储存铁不足，但Hepcidin合成受抑制程度较前减弱。ID期仅有储存铁不足，机体铁缺乏较IDA和IDE改善，抑制Hepcidin合成的因素进一步减少。本研究结果显示IDA组Hepcidin低于ID/IDE组（校正后*P*＝0.005）和正常对照组（校正后*P*<0.001），甚至在IDA患者中也观察到更严重的缺铁伴有更低水平的Hepcidin；ID/IDE组Hepcidin低于正常对照组但差异无统计学意义（校正后*P*＝0.220），说明从IDA到ID/IDE阶段口服铁吸收相对减缓。相关性分析也表明SF为Hepcidin的独立影响因素，随着铁缺乏程度减轻，Hepcidin逐渐回升，铁吸收水平较前下降。提示铁缺乏程度影响铁吸收，随着贫血纠正和铁缺乏改善，进一步补足储存铁将非常缓慢，这与临床IDA口服补铁改善贫血较为容易，但补充储存铁较为困难，且极易IDA复发，ID、IDE静脉补铁优于口服补铁的结果相一致[13]。

OIAT测定C2–C0也用于评估铁吸收[14]–[15]，但研究多限于评估铁缺乏是否存在胃肠吸收因素以及需否更改给药途径，并未在胃肠道功能正常的单纯铁缺乏患者中明确缺铁状态本身对铁吸收的影响。

我们仅纳入摄入不足或失血过多引起的单纯铁缺乏患者，评估不同程度铁缺乏的胃肠道铁吸收，结果显示IDA、ID/IDE、正常对照组C2差异无统计学意义（*P*＝0.147）。OIAT使用固定口服铁剂量，虽然增加口服铁剂量可能提高C2水平，但对于TSAT2已高达90％以上的患者，因体内转铁蛋白已经饱和，继续增加口服铁剂量也不能提升C2。本研究中，正常对照及铁缺乏患者OIAT使用0.3 g硫酸亚铁部分患者2 h TSAT达90％以上，因而我们认为该口服剂量是合适的，无进一步提高口服剂量的必要。

C2–C0为口服铁剂后2 h血清铁升高的绝对值，可用于筛查口服铁吸收不良患者以便尽早更换治疗剂型及用药途径[14],[16]–[20]。我们结果显示正常对照组C2–C0低于铁缺乏症组（*P*＝0.044），提示铁缺乏患者胃肠吸收增加，因为各组C2差异并无统计学意义，故我们认为这主要与患者C0差异有关。正常对照组、IDA、ID/IDE组间C2–C0差异无统计学意义（*P*＝0.13），因而该方法用于进一步区分不同缺铁程度患者胃肠铁吸收并不理想。OIAT以口服铁剂后能够达到的最大血清铁水平与基线值差反映胃肠道铁吸收有否异常是合适的，用于区分不同缺铁程度下铁吸收的差异则是不合适的。

铁缺乏程度影响胃肠道铁吸收，随着缺铁程度的减轻，Hepcidin增高，胃肠铁吸收逐渐减少。血浆Hepcidin和OIAT均能反映胃肠铁吸收，但仅有前者可进一步区分不同缺铁程度铁吸收的差异。
